# Piecewise quadratic neuron model: A tool for close-to-biology spiking neuronal network simulation on dedicated hardware

**DOI:** 10.3389/fnins.2022.1069133

**Published:** 2023-01-09

**Authors:** Takuya Nanami, Takashi Kohno

**Affiliations:** Institute of Industrial Science, The University of Tokyo, Tokyo, Japan

**Keywords:** silicon neuronal network, silicon neuron, spiking neuron model, FPGA, PQN model

## Abstract

Spiking neuron models simulate neuronal activities and allow us to analyze and reproduce the information processing of the nervous system. However, ionic-conductance models, which can faithfully reproduce neuronal activities, require a huge computational cost, while integral-firing models, which are computationally inexpensive, have some difficulties in reproducing neuronal activities. Here we propose a Piecewise Quadratic Neuron (PQN) model based on a qualitative modeling approach that aims to reproduce only the key dynamics behind neuronal activities. We demonstrate that PQN models can accurately reproduce the responses of ionic-conductance models of major neuronal classes to stimulus inputs of various magnitudes. In addition, the PQN model is designed to support the efficient implementation on digital arithmetic circuits for use as silicon neurons, and we confirm that the PQN model consumes much fewer circuit resources than the ionic-conductance models. This model intends to serve as a tool for building a large-scale closer-to-biology spiking neural network.

## 1. Introduction

Spiking neural network (SNN) models can reproduce the information processing of the nervous system at the cellular and synaptic level, enabling us to analyze and understand the brain's information processing and to realize the brain's intelligence for engineering applications. While large-scale simulations of SNN models using general-purpose computers require extensive computational facilities and are slow in simulation speed, the silicon neuronal networks (SiNNs), which are electronic circuit systems specifically designed to simulate SNN models, enable highly power-efficient and high-speed simulation. A main element of SiNNs is the silicon neuron that simulates neuronal activities by using the spiking neuron model. Silicon neurons using analog circuits (Schemmel et al., 2010; Arthur and Boahen, 2011; Grassia et al., 2011; Brink et al., 2013; Qiao et al., 2015; Kohno and Aihara, 2016; Moradi et al., 2018; Rubino et al., 2021; Schemmel et al., 2021) show very high power efficiency, but have technical hurdles including fabrication mismatch and temperature dependence. In contrast, silicon neurons using digital circuits are far less sensitive to these factors. Although they tend to consume higher power than analog silicon neurons, they are more portable, easy-to-operate, and highly integratable. Large-scale networks have been built using digital application-specific integrated circuits (ASICs) (Merolla et al., 2014; Davies et al., 2018). In addition, field-programmable gate arrays (FPGAs) have also been used (Thomas and Luk, 2009; Cassidy et al., 2011, 2013; Li et al., 2012; Khoyratee et al., 2019) due to their ease of implementation and low cost.

**Figure 1:**
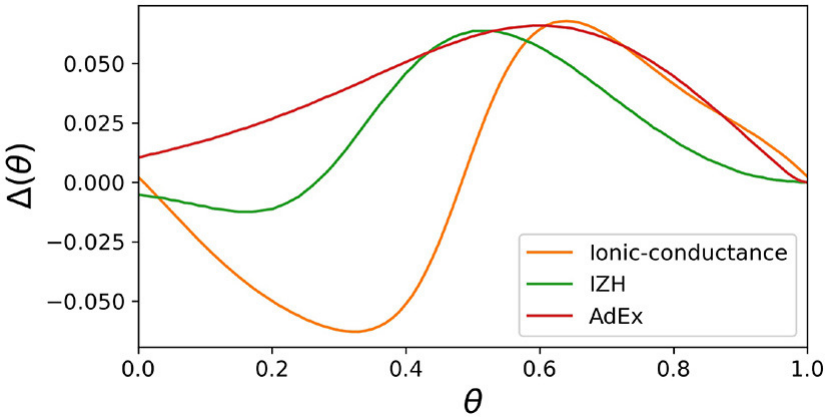
Phase-resetting curve of spiking neuron models. The horizontal axis represents the phase at which the pulse stimulus was given, and the vertical axis represents how much the phase was shifted by the pulse stimulus.

In both SNN models and SiNNs, a variety of spiking neuron models have been used due to the trade-off between the reproducibility of neuronal activities and computational efficiency. The ionic-conductance neuron models (Hodgkin and Huxley, 1952; Plant, 1981; Wang, 1993; SCHUTTER and BOWER, 1994; Pospischil et al., 2008) describe neuronal dynamics at the level of each ion channel and can replicate arbitrary electrophysiological properties. The nervous system contains a wide variety of neuronal classes, each with different electrophysiological properties, which are reported (Cunningham et al., 2004; Benda et al., 2005; Grillner, 2007) to play an essential role in information processing. Therefore ionic-conductance models are used in SNN models (Markram et al., 2015; Bezaire et al., 2016; Ecker et al., 2020) that attempt to fully replicate certain brain regions. However, they consume enormous resources in circuit implementation, making their application to large-scale SiNNs difficult. In contrast, the Leaky integrate-and-fire (LIF) model is one of the simplest spiking neuron models and reproduces only simplified features such as the perturbation by stimulus inputs over time and convergence to the resting potential by the leak current. The LIF model approximates the neuronal spiking process by resetting the state variables and is therefore very computationally inexpensive. It is used for SiNNs (Cassidy et al., 2011; Merolla et al., 2014; Davies et al., 2018) that intend to realize large-scale networks with limited power consumption. In addition, the Izhikevich (IZH) model (Izhikevich, 2003) and the adaptive exponential I&F (AdEx) model (Brette and Gerstner, 2005), which are extensions of the LIF model, similarly approximate the spiking process by resetting the state variables. They have also been used in many SNN models (Thomas and Luk, 2009; Schemmel et al., 2010; Cassidy et al., 2013; Qiao et al., 2015; Moradi et al., 2018; Rubino et al., 2021; Schemmel et al., 2021) because they cover several neuronal classes, although they require a little more computational cost than the LIF model due to their squared and exponential term. In these integrate-and-fire-based (I&F-based) models, resetting the state variables reduces their computational cost but also compromises the reproducibility of neuronal activities. For example, it is known that the amplitude of spikes is gradedly dependent on the stimulus in some regions in the nervous system (Alle and Geiger, 2006), which is referred to as graded response. It was shown theoretically (Rinzel and Ermentrout, 1998) that the Class *II* in the Hodgkin's classification (Hodgkin, 1948) can exhibit the graded response. However, the I&F-based models, even in their Class *II* modes, cannot realize the graded response because it assumes the spike as a stereotyped event. Another example is the phase-resetting curve (PRC) (Rinzel and Ermentrout, 1998), which represents how the phase of a neuron in an oscillatory spiking state changes depending on the timing of the pulse stimulus. The Class *II* modes of the IZH and AdEx models reproduce the discontinuous frequency-current curve, but the shape of their PRC is not the standard Type *II*, which is typical for the Class *II* (Figure 1). Because neurons with the Type *II* PRC tend to promote synchronous firing (Hansel et al., 1995), it might be important to model the synchronous activities in the brain. As shown in Figure 1, the Class *II* mode of the ionic-conductance model has a biphasic (Type *II*) PRC, while the PRC of the AdEx model is monophasic. The Class *II* mode of the IZH model has a very small negative part and Δ(θ) is less than 0 when θ = 0. Furthermore, it was reported (Nanami and Kohno, 2016) that the responses of the intrinsically bursting (IB) class of the IZH model to step stimuli are different from those of a typical ionic-conductance model.

A qualitative modeling approach has a long history that starts with the FitzHugh–Nagumo model (FitzHugh, 1961; Nagumo et al., 1962) and was theoretically established by the theory of nonlinear dynamics (Kepler et al., 1992; Rinzel and Ermentrout, 1998; Izhikevich, 2007). The qualitative neuron models (FitzHugh, 1961; Nagumo et al., 1962; Hindmarsh and Rose, 1984; Nanami and Kohno, 2016) reproduce only the key mathematical structures behind neuronal activities. Although they have much less computational cost than the ionic-conductance models, they can faithfully reproduce the dynamics of the spiking process without resetting the state variables. In the FitzHugh–Nagumo model and Hindmarsh–Rose model (Hindmarsh and Rose, 1984), the right-hand sides of the differential equations for each state variable are represented by polynomials of degree 3 or less. The digital spiking silicon neuron (DSSN) model (Nanami and Kohno, 2016) employs quadratic terms with piecewise and step functions to achieve qualitatively equivalent dynamics, and this approximation enables efficient implementation in software and digital arithmetic circuits. In addition, while preceding qualitative models represent a single neuron class, the DSSN model is designed to represent a wide variety of classes by tuning its parameters. The DSSN model covers the regular spiking (RS), fast spiking (FS), low-threshold spiking (LTS), IB, elliptic bursting (EB), and parabolic bursting (PB) classes. In the FS mode, both the Class *I* and Class *II* in Hodgkin's classification are supported. Recently, a general framework of qualitative models described by piece-wise polynomial formula has been proposed (Tikidji-Hamburyan and Colonnese, 2021). This framework, polynomial, piecewise-linear, step (PLS)-framework, provides a generalized method of constructing, describing, and discussing these models. In this framework, the right-hand side of the differential equation is expressed as any combination of polynomial, piecewise-linear, and step functions.

In this study, we propose a Piecewise Quadratic Neuron (PQN) model, a refined version of the DSSN model. Though the DSSN model supports various neuronal classes, its ability to reproduce their dynamical activity for a wide range of stimulus intensity is limited. In addition, a number of parameters have to be fitted to reproduce a wide variety of neuronal dynamics, which makes the fitting difficult. Thus, a scaling parameter for the stimulus input is inserted and six parameters are omitted in the PQN model by assuming that the nullcline of each state variable is continuous and smooth. Since the cellular dynamics corresponding to these nullclines are expected to be continuous and smooth, this assumption is biologically natural. The reduction in the number of parameters leads to a drastic reduction in the explore space in the parameter tuning, facilitating better parameter sets. First, it is demonstrated that the PQN model can reproduce the graded response and the Type *II* PRC typically seen in the Class *II* neurons in the Hodgkin's classification. Next, it is shown that the PQN model reproduces six major neuronal classes, RS, FS, LTS, IB, EP, and PB. Here, we confirm that the PQN model can reproduce the activity of ionic-conductance models of six neuronal classes in response to step inputs of various magnitudes. Although these neuron classes were also supported by the DSSN model, their responses to step inputs with a wide range of intensity are not completely reproduced. In addition, we show that the PQN model consumes much fewer circuit resources than the ionic-conductance model in the FPGA implementation due to its simple equations fitted to the digital arithmetic circuits. Moreover, we provide the python codes for the software simulation and VHDL codes for hardware implementation and they are available at the following links (PQN_pya^1^; PQN_vhdl^2^). Note that the model must be computed using the fixed-point operation with the specified bit-width, 28 bits for Class *II* and 18 bits otherwise, and the Euler's method with the specified time-step width, 1 ms for PB and 0.1 ms otherwise. In Methods section, in addition to the original form of the PQN model, equations using the PLS-framework are also presented for a more comprehensive perspective. This study intends to provide an easy-to-use tool for constructing large-scale and close-to-biology SNN models and SiNNs.

The remainder of this paper is organized as follows: Section 2 describes the details of the model, Section 3 shows the simulation results and comparison, and Section 4 provides a summary and future perspectives.

## 2. Methods

### 2.1. Equations of the PQN model

In order to efficiently reproduce a variety of neuronal classes, each with different dynamics, the PQN model has four variations. The first one is the two-variable PQN model, which is the basic form of the other variations. It supports the Class *II* mode in the Hodgkin's classification. Its equations are as follows:


(1) $$\begin{array}{l} \frac{dv}{dt}=\frac{\phi }{\tau }\left(f\left(v\right)-n+I_{0}+kI_{\text{stim}}\right), \end{array}$$

(2) $$\begin{array}{l} \frac{dn}{dt}=\frac{1}{\tau }\left(g\left(v\right)-n\right), \end{array}$$

(3) $$\begin{array}{l} f\left(v\right)=\{\begin{array}{c} a_{fn}{\left(v-b_{fn}\right)}^{2}+c_{fn}\;\left(v<0\right)\\ a_{fp}{\left(v-b_{fp}\right)}^{2}+c_{fp}\;\left(v\geq 0\right), \end{array} \end{array}$$

(4) $$\begin{array}{l} g\left(v\right)=\{\begin{array}{c} a_{gn}{\left(v-b_{gn}\right)}^{2}+c_{gn}\;\left(v<r_{g}\right)\\ a_{gp}{\left(v-b_{gp}\right)}^{2}+c_{gp}\;\left(v\geq r_{g}\right), \end{array} \end{array}$$

(5) $$\begin{array}{l} b_{fp}=\frac{a_{fn}b_{fn}}{a_{fp}}, \end{array}$$

(6) $$\begin{array}{l} c_{fp}=a_{fn}b_{fn}^{2}+c_{fn}-a_{fp}b_{fp}^{2}, \end{array}$$

(7) $$\begin{array}{l} b_{gp}=r_{g}-\frac{a_{gn}\left(r_{g}-b_{gn}\right)}{a_{gp}}, \end{array}$$

(8) $$\begin{array}{l} c_{gp}=a_{gn}{\left(r_{g}-b_{gn}\right)}^{2}+c_{gn}-a_{gp}{\left(r_{g}-b_{gp}\right)}^{2}, \end{array}$$

where *v* and *n* represent the membrane potential and recovery variable, respectively. Parameter *I*_0_ is a bias constant. *I*_stim_ is the stimulus input and *k* is its scaling parameter. The parameters τ and ϕ determine the time constants of the variables. The parameters *r*_*g*_, *a*_*x*_, *b*_*x*_, and *c*_*x*_, where *x* is *fn*, *fp*, *gn*, or *gp*, are constants that determine the nullclines of the variables. Constants *b*_*fp*_, *c*_*fp*_, *b*_*gp*_, and *c*_*gp*_, are determined by other parameters such that the nullclines are continuous and smooth. All of the variables and parameters are purely abstract with no physical units. The differences from the DSSN model are that the four constants, *b*_*fp*_, *c*_*fp*_, *b*_*gp*_, and *c*_*gp*_, are determined by other parameters to ensure continuity and smoothness, and that the parameter *k*, which scales the stimulus input, is inserted. The ionic-conductance models generally require three or more state variables for the calculation of the spiking process, and the differential equations include a quartic term and exponential functions (Hodgkin and Huxley, 1952; Pospischil et al., 2008). On the other hand, the PQN model requires only two variables with quadratic terms and can be implemented with less computational cost in software and hardware implementations.

The second one is the three-variable PQN model. A new variable *q* is introduced to support the RS, FS, and EB classes. Its equations are given by


(9) $$\begin{array}{l} \frac{dv}{dt}=\frac{\phi }{\tau }\left(f\left(v\right)-n-q+I_{0}+kI_{\text{stim}}\right), \end{array}$$

(10) $$\begin{array}{l} \frac{dn}{dt}=\frac{1}{\tau }\left(g\left(v\right)-n\right), \end{array}$$

(11) $$\begin{array}{l} \frac{dq}{dt}=\frac{\epsilon_{q}}{\tau }\left(h\left(v\right)-q\right), \end{array}$$

(12) $$\begin{array}{l} h\left(v\right)=\{\begin{array}{l} a_{hn}{\left(v-b_{hn}\right)}^{2}+c_{hn}\;\left(v<r_{h}\right)\\ a_{hp}{\left(v-b_{hp}\right)}^{2}+c_{hp}\;\left(v\geq r_{h}\right), \end{array} \end{array}$$

(13) $$\begin{array}{l} b_{hp}=r_{h}-\frac{a_{hn}\left(r_{h}-b_{hn}\right)}{a_{hp}}, \end{array}$$

(14) $$\begin{array}{l} c_{hp}=a_{hn}{\left(r_{h}-b_{hn}\right)}^{2}+c_{hn}-a_{hp}{\left(r_{h}-b_{hp}\right)}^{2}, \end{array}$$

where *q* represents the slow variable. The parameter ϵ_*q*_ determine its time constants and parameters *r*_*h*_, *a*_*x*_, *b*_*x*_, and *c*_*x*_, where *x* is *hn* or *hp*, determine its nullcline. Constants *b*_*hp*_ and *c*_*hp*_ are determined by other parameters such that the nullcline is continuous and smooth. Note that Equation (9) is the same as Equation (1) except that the new variable *q* is inserted. Equation (10) is the same as Equation (2). The difference from the DSSN model is that the constants *b*_*fp*_, *c*_*fp*_, *b*_*gp*_, *c*_*gp*_, *b*_*hp*_, and *c*_*hp*_ are defined by other parameters and *k* is inserted.

The third one is the four-variable PQN model. Another variable *u* is introduced to support the PB class. Its equations are


(15) $$\begin{array}{l} \frac{dv}{dt}=\frac{\phi }{\tau }\left(f\left(v\right)-n-q+u+I_{0}+kI_{\text{stim}}\right), \end{array}$$

(16) $$\begin{array}{l} \frac{dn}{dt}=\frac{1}{\tau }\left(g\left(v\right)-n\right), \end{array}$$

(17) $$\begin{array}{l} \frac{dq}{dt}=\frac{\epsilon_{q}}{\tau }\left(h\left(v\right)-q\right), \end{array}$$

(18) $$\begin{array}{l} \frac{du}{dt}=\frac{\epsilon_{u}}{\tau }\left(v+v_{0}-\alpha_{u}u\right), \end{array}$$

where *u* is the slow variable and the slow subsystem consisting of *u* and *q* is capable of producing oscillations. The parameter ϵ_*u*_ determines the time constant of *u*. The parameters *v*_0_ and α_*u*_ determine the nullcline of *u*, respectively. Note that Equation (15) is the same as Equation (9) except that variable *u* is incorporated. Equations (16) and (17) are shared with the three-variable PQN model. The differences from the DSSN model are exactly the same as those in the three-variable mode.

The final variation is the extended four-variable PQN model, which supports the LTS and IB classes. The only difference to the four-variable PQN model is that the time constant of *n* is dependent on *u*. Its equations are given by


(19) $$\begin{array}{l} \frac{dv}{dt}=\frac{\phi }{\tau }\left(f\left(v\right)-n-q+I_{0}+kI_{\text{stim}}\right), \end{array}$$

(20) $$\begin{array}{l} \frac{dn}{dt}=\frac{\eta \left(u\right)}{\tau }\left(g\left(v\right)-n\right), \end{array}$$

(21) $$\begin{array}{l} \frac{dq}{dt}=\frac{\epsilon_{q}}{\tau }\left(h\left(v\right)-q\right), \end{array}$$

(22) $$\begin{array}{l} \frac{du}{dt}=\frac{\epsilon_{u}}{\tau }\left(v+v_{0}-\alpha_{u}u\right), \end{array}$$

(23) $$\begin{array}{l} \eta \left(u\right)=\{\begin{array}{l} \eta_{0}\;\left(u<r_{u}\right)\\ \eta_{1}\;\left(u\geq r_{u}\right), \end{array} \end{array}$$

where *u* is a state variable with a large time constant that varies the structure of the fast subsystem consisting of *v* and *n*. The η controls the time constant of *n*, whose value is determined to be η_0_ or η_1_ depending on the value of *u* and a threshold *r*_*u*_. In the DSSN model, the LTS class belongs to the three-variable mode. In the PQN model, it is included in the extended four-variable model to reproduce the rebound bursting more faithfully. In the extended four-variable model, the structure of the fast subsystem consisting of *v* and *n* is shifted depending on the change in the time constant of the state variable. In the DSSN model, the time constant of *v* is varied by *u*, whereas in the PQN model, the time constant of *n* is varied instead to avoid increasing the calculation process for *v*.

These differential equations are numerically integrated with Euler's method, whose time steps are 1 ms for the PB class and 0.1 ms for the other classes. The neuronal parameters are fitted so that the model reproduces the properties of each class as accurately as possible and consumes as few circuit resources as possible in the digital arithmetic circuit implementation. The parameters are determined by a semi-automatic optimization method developed previously, the detailed protocol of which is described in Nanami et al. (2017, 2018). Since the synaptic current is not fitted in this study, the synaptic parameters α and β are the same values used in the previous study (Li et al., 2012). The neuronal parameters of all classes are listed in the Supplementary material.

In the PLS framework, neuron models are described by a combination of core functions P, L, and S, which represent polynomial, piecewise-linear, and step functions, respectively. For example, core functions *P*_1_, *L*_1_, and *S*_1_ are defined as follows:


(24) $$\begin{array}{l} P_{1}\left(x,x_{0}\right)=x_{0}-x, \end{array}$$

(25) $$\begin{array}{l} L_{1}\left(x,x_{0},x_{1},x_{2}\right)=x_{1}+x_{2}\left(x-x_{0}\right), \end{array}$$

(26) $$\begin{array}{l} S_{1}\left(x,x_{0},x_{1},x_{2}\right)=\{\begin{array}{l} x_{1}\;\left(x<x_{0}\right)\\ x_{2}\;\left(x\geq x_{0}\right), \end{array} \end{array}$$

The Class II mode of the PQN model in the PLS-framework is given by


(27) $$\begin{array}{l} \frac{\tau }{\theta }\frac{d\upsilon }{dt}=S_{1}(\upsilon ,0,a_{fn}{\left(P_{1}\left(\upsilon ,b_{fn}\right)\right)}^{2}\\ \;+c_{fn},a_{fp}{\left(P_{1}\left(\upsilon ,b_{fp}\right)\right)}^{2}+c_{fp})-n+k\;I_{stim}, \end{array}$$

(28) $$\begin{array}{l} \tau \frac{dn}{dt}=S_{1}(\upsilon ,r_{g},a_{gn}{\left(P_{1}\left(\upsilon ,b_{gn}\right)\right)}^{2}\\ \;+c_{gn},a_{gp}{\left(P_{1}\left(\upsilon ,b_{gp}\right)\right)}^{2}+c_{gp})-n. \end{array}$$

Equations of the RS, FS, and EB classes are given by


(29) $$\begin{array}{l} \frac{\tau }{\theta }\frac{d\upsilon }{dt}=S_{1}\left(\upsilon ,0,a_{fn}{\left(P_{1}\left(\upsilon ,b_{fn}\right)\right)}^{2}+c_{fn},a_{fp}{\left(P_{1}\left(\upsilon ,b_{fp}\right)\right)}^{2}+c_{fp}\right)\\ \;-n-q+k\;I_{stim}, \end{array}$$

(30) $$\begin{array}{l} \tau \frac{dn}{dt}=S_{1}(\upsilon ,r_{g},a_{gn}{\left(P_{1}\left(\upsilon ,b_{gn}\right)\right)}^{2}\\ \;+c_{gn},a_{gp}{\left(P_{1}\left(\upsilon ,b_{gp}\right)\right)}^{2}+c_{gp})-n, \end{array}$$

(31) $$\begin{array}{l} \frac{\tau }{\epsilon_{q}}\frac{dq}{dt}=S_{1}(\upsilon ,r_{h},a_{hn}{\left(P_{1}\left(\upsilon ,b_{hn}\right)\right)}^{2}\\ \;+c_{hn},a_{hp}{\left(P_{1}\left(\upsilon ,b_{hp}\right)\right)}^{2}+c_{hp})-q, \end{array}$$

and equations of the PB classes are given by


(32) $$\begin{array}{l} \frac{\tau }{\theta }\frac{dv}{dt}=S_{1}(\upsilon ,0,a_{fn}{\left(P_{1}\left(\upsilon ,b_{fn}\right)\right)}^{2}+c_{fn},a_{fp}{\left(P_{1}\left(\upsilon ,b_{fp}\right)\right)}^{2}\\ \;+c_{fp})-n-q+u+k\;I_{stim}, \end{array}$$

(33) $$\begin{array}{l} \tau \frac{dn}{dt}=S_{1}(\upsilon ,r_{g},a_{gn}{\left(P_{1}\left(\upsilon ,b_{gn}\right)\right)}^{2}\\ \;+c_{gn},a_{gp}{\left(P_{1}\left(\upsilon ,b_{gp}\right)\right)}^{2}+c_{gp})-n, \end{array}$$

(34) $$\begin{array}{l} \frac{\tau }{\epsilon_{q}}\frac{dq}{dt}=S_{1}(\upsilon ,r_{h},a_{hn}{\left(P_{1}\left(\upsilon ,b_{hn}\right)\right)}^{2}\\ \;+c_{hn},a_{hp}{\left(P_{1}\left(\upsilon ,b_{hp}\right)\right)}^{2}+c_{hp})-q, \end{array}$$

(35) $$\begin{array}{l} \frac{\tau }{\epsilon_{u}\alpha_{u}}\frac{du}{dt}=L_{1}\left(\upsilon ,\;0,\frac{\upsilon_{0}}{\alpha_{u}},\frac{1}{\alpha_{u}}\right)-u. \end{array}$$

Equations of the LTS and IB classes are written using the step function as follows:


(36) $$\begin{array}{l} \frac{\tau }{\theta }\frac{dv}{dt}=S_{1}(\upsilon ,0,a_{fn}{\left(P_{1}\left(\upsilon ,b_{fn}\right)\right)}^{2}\\ \;+c_{fn},a_{fp}{\left(P_{1}\left(\upsilon ,b_{fp}\right)\right)}^{2}+c_{fp})\\ \;-n-q+k\;I_{stim}, \end{array}$$

(37) $$\begin{array}{l} S1\left(u,r_{u},\frac{\tau }{\eta_{0}},\frac{\tau }{\eta_{1}}\right)\frac{dn}{dt}=S_{1}(\upsilon ,r_{g},a_{gn}{\left(P_{1}\left(\upsilon ,b_{gn}\right)\right)}^{2}+c_{gn},\\ \;a_{gp}{\left(P_{1}\left(\upsilon ,b_{gp}\right)\right)}^{2}+c_{gp})-n, \end{array}$$

(38) $$\begin{array}{l} \frac{\tau }{\epsilon_{q}}\frac{dq}{dt}=S_{1}(\upsilon ,r_{h},a_{hn}{\left(P_{1}\left(\upsilon ,b_{hn}\right)\right)}^{2}+c_{hn},\\ \;a_{hp}{\left(P_{1}\left(\upsilon ,b_{hp}\right)\right)}^{2}+c_{hp})-q, \end{array}$$

(39) $$\begin{array}{l} \frac{\tau }{\epsilon_{u}\alpha_{u}}\frac{du}{dt}=L_{1}\left(\upsilon ,0,\frac{\upsilon_{0}}{\alpha_{u}},\frac{1}{\alpha_{u}}\right)-u. \end{array}$$

### 2.2. Implementations of the PQN model

**Figure 2:**
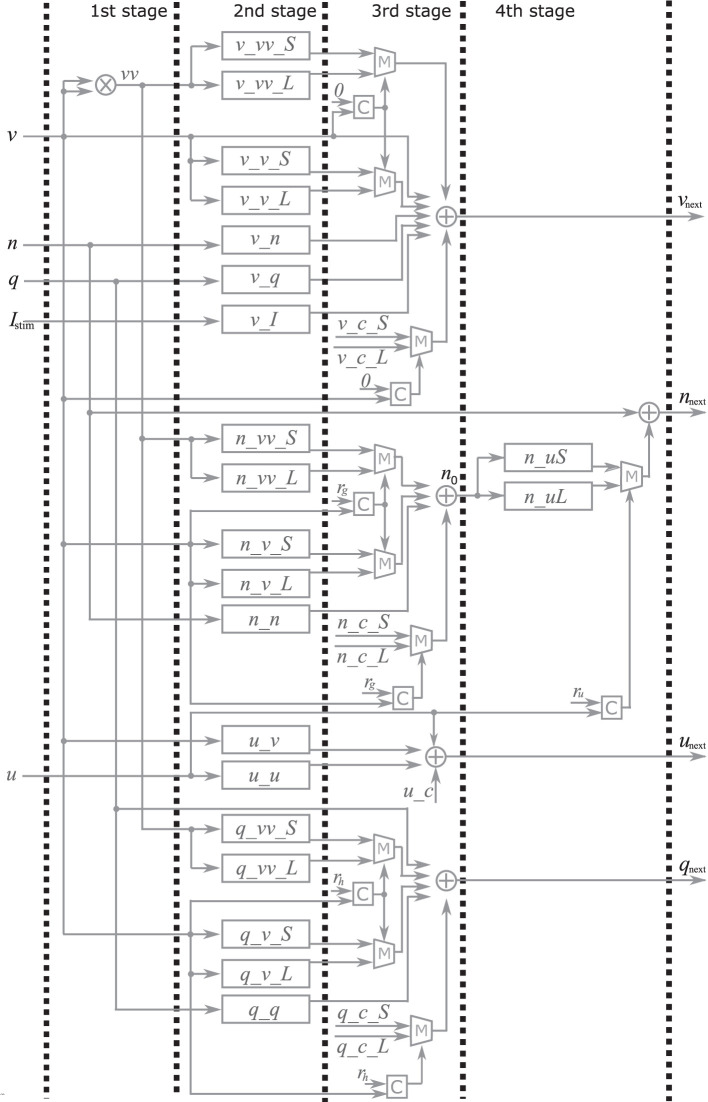
Block diagram of the PQN engine for the IB and LTS mode. The symbols ×, +, *M* and *C* represent multipliers, adders, multiplexers, and comparators, respectively. The *v*, *n*, *q*, *u* and *I*_stim_ are input for the PQN engine. The output *v*_*next*_, *n*_*next*_, *q*_*next*_, and *u*_*next*_ are values of the state variables at the next time step.

**Figure 3:**
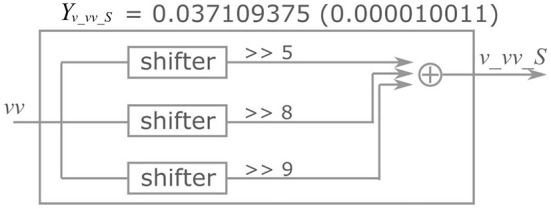
An example of the multiplication of a state variable and a coefficient.

Here, the implementation of the extended four-variable model, the most complex variant, is explained. Figure 2 shows the block diagram of the PQN engine, which calculates the state variables at the next time step. The symbols ×, +, *M*, and *C* in the figure represent multipliers, adders, multiplexers, and comparators, respectively. The *vv* is a square of *v* and the *n*_0 is an intermediate result of calculation for *n*_next_. The *v*_*c*, *n*_*c*, *q*_*c*, *u*_*c*, and *s*_*c*_*L*, are constants, and they are calculated in advance and stored. The signals *v*_*vv*_*S*, *v*_*vv*_*L*, *v*_*v*_*S*, *v*_*v*_*L*, *v*_*n*, *v*_*q*, *v*_*I*, *n*_*uS*, *n*_*uL*, *n*_*vv*_*S*, *n*_*vv*_*L*, *n*_*v*_*S*, *n*_*v*_*L*, *n*_*n*, *q*_*vv*_*S*, *q*_*vv*_*L*, *q*_*v*_*S*, *q*_*v*_*L*, *q*_*q*, *u*_*v*, and *u*_*u* are the intermediate results of the calculation. They are calculated by the multiplication of a signal and a coefficient, which is implemented by shifters and adders. For example, Figure 3 shows the calculation of the signal *v*_*vv*_*S*. If *Y*_*v*_*vv*_*S*_ is 0.037109375, and its binary representation is 0.000010011. Therefore, calculating the sum of the sixth, fifth, and ninth right-shift operations on the square of *v* is performed. The results of the calculations are stored in registers for pipelined design. Details of the signals, coefficients, and constants are shown in Supplementary material.

In the first stage, the square of *v* is calculated using the multiplier. The second stage involves the multiplication of the variables and coefficients. In the third stage, the result of the calculation of *v*_next_, *q*_next_, *u*_next_is obtained, In the fourth stage, the value of *s*_next_ is determined based on the *s* and *v*_next_ the value of *n*_next_ is determined based on the *u* and *n*_0_.

**Figure 4:**
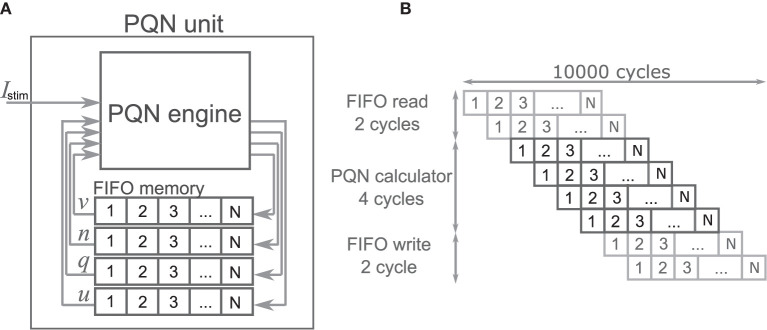
The structure and clock cycle of the PQN unit. **(A)** The internal structure of the PQN unit. The state variables are stored in the FIFO memory and sent to the PQN engine in turn. **(B)** The clock cycle of the PQN unit.

The PQN unit is a complex of a PQN engine and registers for the state variables (Figure 4A). The registers are configured as FIFO memory and the values are in turn passed to the PQN engine. Here, N represents the number of neurons that one PQN unit computes. The values of the state variables for the next time step are returned to the FIFO memory and stored. Figure 4B shows the pipeline usage of the PQN unit. In IB and LTS modes, the time step is 0.1 ms. To perform the real-time simulation at 100 MHz clock, 10,000 cycles can be consumed to update all state variables. Since the PQN engine takes 4 cycles and reads and writes of the FIFO require 3 clocks, the N becomes 9993.

Since multiplications between a coefficient and state variable are realized by shifters and adders, the values of the parameters affect the circuit resources. Each additional number of ones in the binary representation of the coefficients consumes an additional pair of shifters and adders.

Except for the Class *II* mode, all state variables are expressed in an 18-bit fixed-point with an 8-bit integer part. In the Class *II* mode with the 18-bit fixed-point, the PRC is biphasic but not smooth, so we used a 28-bit fixed-point with an 8-bit integer part representation.

## 3. Results

### 3.1. Simulation results of the class *II* mode

**Figure 5:**
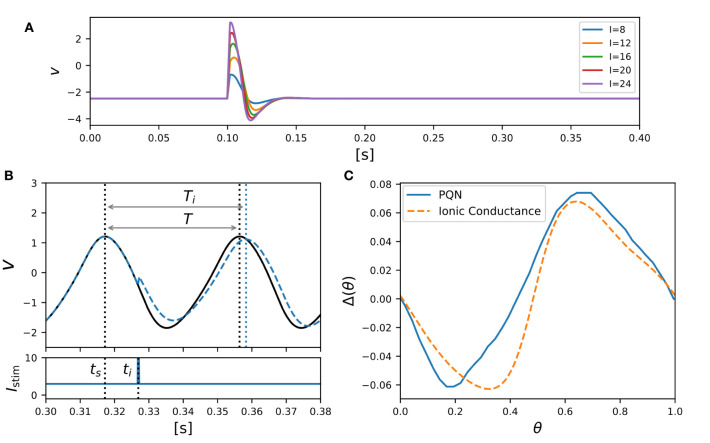
PQN model of Class *II* mode in the Hodgkin's classification. **(A)** Graded responses to pulse stimuli. Pulse stimuli of various magnitudes were given between 0.1 and 0.102 [s]. **(B)** Phase changes induced by the pulse stimulus. **(C)** PRCs of the PQN model and the ionic-conductance model. The horizontal axis represents the phase at which the pulse stimulus was given, and the vertical axis represents how much the phase was shifted by the pulse stimulus.

Class *II* mode in the Hodgkin's classification is known (Rinzel and Ermentrout, 1998) to show the graded response to pulsed stimuli and has the biphasic PRC. Figure 5A shows simulation results of the PQN model in response to five pulse stimuli of different magnitudes. The maximum amplitude of the spike increases as the magnitude of the pulse stimulus increases, which is referred to as the graded response.

The PRC was evaluated by measuring phase shift while varying the phase of the pulse stimulus. The phase of the pulse stimulus θ and phase shift Δ(θ) are defined as follows:


(40) $$\begin{array}{l} \theta =\frac{t_{i}-t_{s}}{T}, \end{array}$$

(41) $$\begin{array}{l} \Delta \left(\theta \right)=\frac{T-T_{i}}{T}, \end{array}$$

where *T* represents the time length of the oscillatory spiking cycle, and *t*_*s*_ and *t*_*i*_ are the time of the beginning of the cycle and the time when the pulse stimulus was given, respectively (Figure 5B). *T*_*i*_ represents the length of the resulting cycle shifted by the pulse stimulus. Figure 5C shows the resulting PRC, and it exhibits the standard Type *II* as well as the ionic-conductance model.

### 3.2. Simulation results of the RS, FS, LTS, IB, EB, and PB modes

The target models for the RS, FS, LTS, and IB classes are the ionic-conductance models given in Pospischil et al. (2008). For the EB and PB classes, the ionic-conductance models shown in Plant (1981) and Wang (1993) are chosen, respectively. For the RS class, we reproduced an excitatory cell and an inhibitory cell shown in Pospischil et al. (2008), and thus we prepared seven PQN model parameter sets in total. All the results were obtained using a Xilinx Artix-7 XC7A35T FPGA on a Digilent cmod-a7 board. The circuit was developed using Xilinx Vivado 2018.3 software. The clock signal of the FPGA is 100 MHz. The input current was given from the PC to the PQN unit on the FPGA via serial communication, and the value of the membrane potential *v* of the PQN unit was sent back to the PC. For comparison, we also prepared simulation results of DSSN models for each class; the DSSN model presented in Nanami et al. (2017, 2018) for the RS, FS, LTS, and IB classes, and Nanami et al. (2016) for the EB and PB classes. The DSSN models and ionic-conductance models were simulated on a PC using the python software.

**Figure 6:**
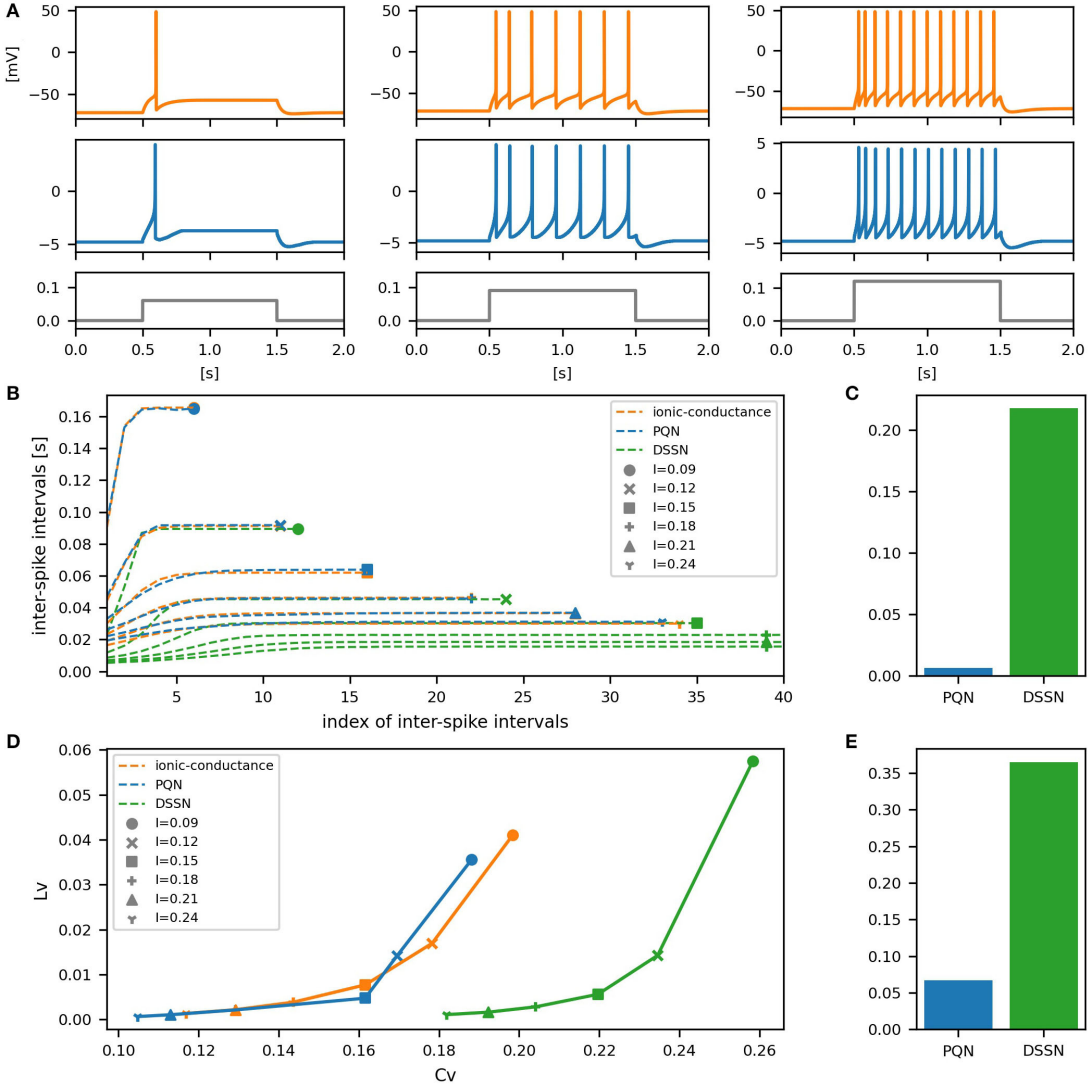
Waveforms and spiking properties in excitatory RS mode. **(A)** Waveforms of the ionic-conductance model (orange) and the PQN model (blue). Gray lines represent stimulus inputs, whose unit in the ionic-conductance model is nA. In the PQN model, they have no physical unit. **(B)** The transitions of ISIs are shown while increasing the magnitude of the stimulus input. The horizontal axis represents the index of the ISIs, and the vertical axis represents the magnitude of the ISIs. Orange, blue, and green lines represent the ionic-conductance, PQN, and DSSN models, respectively. Markers were plotted to show the corresponding magnitude of the stimulus input. **(C)** The mean square errors of the PQN model and the DSSN model with the ionic-conductance model over the data points shown in **(B)**. **(D)** Values of the *C*_*V*_ and *L*_*V*_ were calculated from the waveforms and plotted while varying the magnitude of the stimulus inputs. The waveforms used here are the same as that used in **(B)**. **(E)** The mean square errors of the PQN model and the DSSN model with the ionic-conductance model over the data points shown in **(D)**.

**Figure 7:**
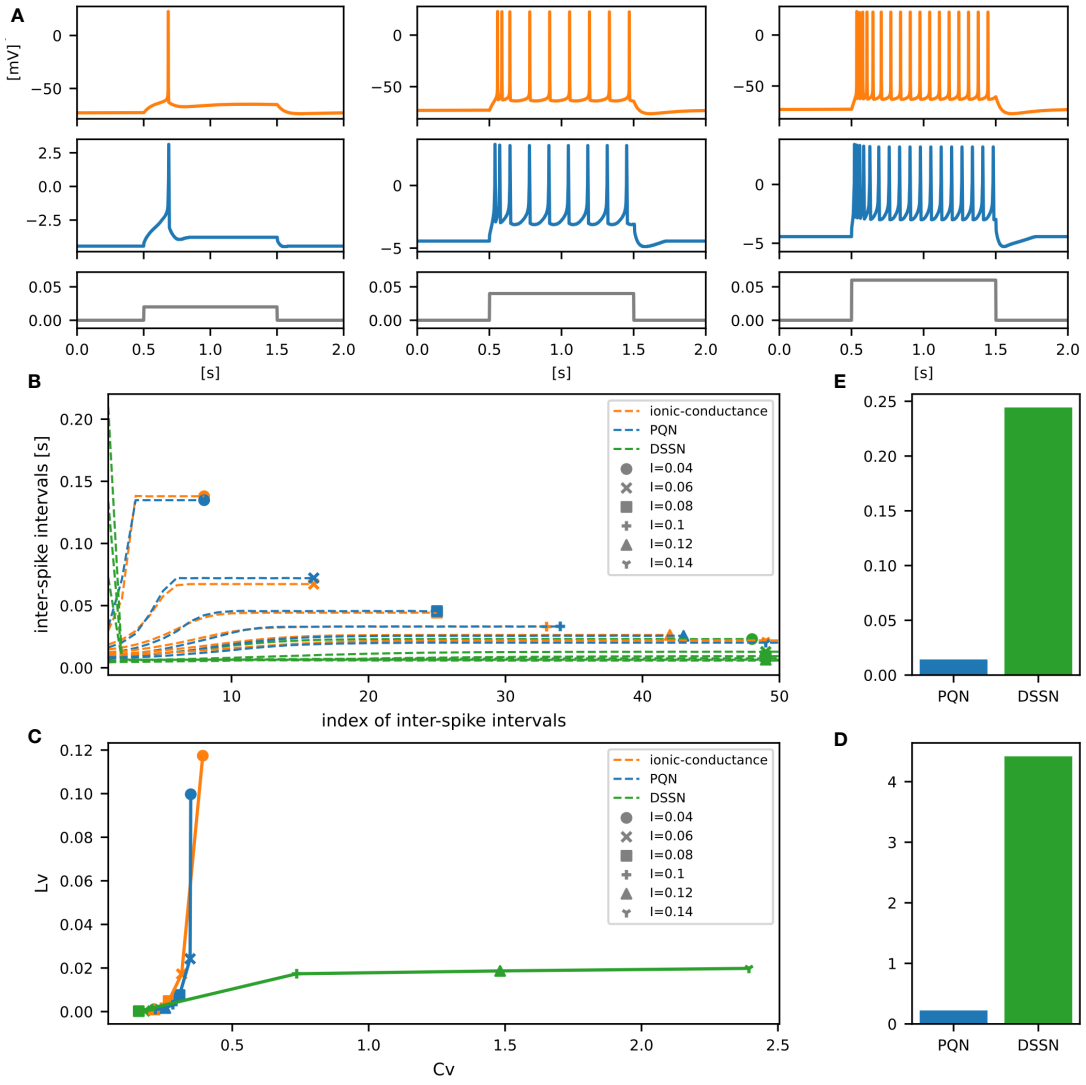
Waveforms and spiking properties in inhibitory RS mode. **(A)** Waveforms of the ionic-conductance model (orange) and the PQN model (blue). Gray lines represent stimulus inputs. **(B)** The transitions of ISIs are shown while increasing the magnitude of the stimulus input. **(C)** The mean square errors of the PQN model and the DSSN model with the ionic-conductance model over the data points shown in **(B)**. **(D)** Values of the *C*_*V*_ and *L*_*V*_ were calculated from the waveforms and plotted while varying the magnitude of the stimulus inputs. The waveforms used here are the same as that used in **(B)**. **(E)** The mean square errors of the PQN model and the DSSN model with the ionic-conductance model over the data points shown in **(D)**.

**Figure 8:**
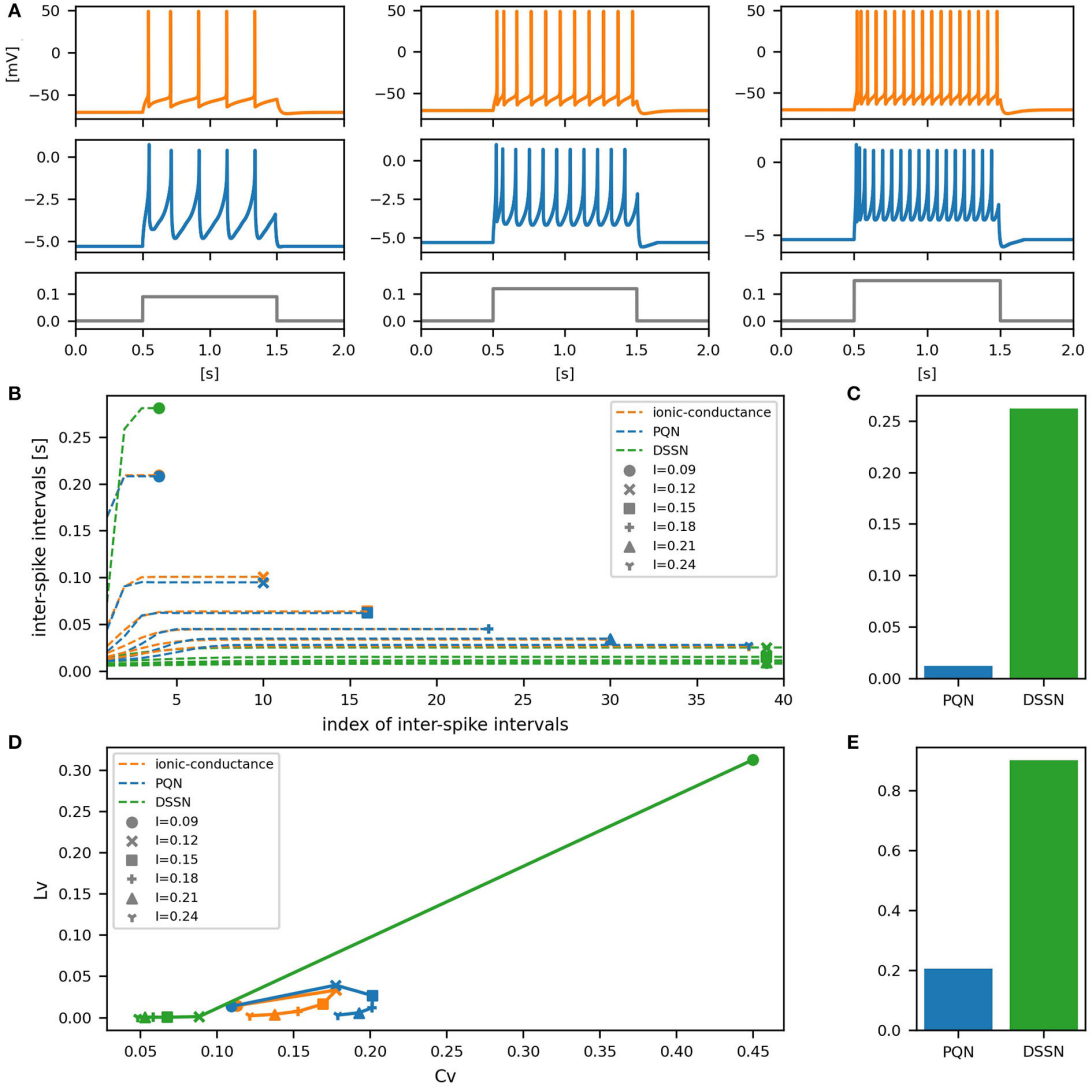
Waveforms and spiking properties in FS mode. **(A)** Waveforms of the ionic-conductance model (orange) and the PQN model (blue). Gray lines represent stimulus inputs. **(B)** The transitions of ISIs are shown while increasing the magnitude of the stimulus input. **(C)** The mean square errors of the PQN model and the DSSN model with the ionic-conductance model over the data points shown in **(B)**. **(D)** Values of the *C*_*V*_ and *L*_*V*_ were calculated from the waveforms and plotted while varying the magnitude of the stimulus inputs. The waveforms used here are the same as that used in **(B)**. **(E)** The mean square errors of the PQN model and the DSSN model with the ionic-conductance model over the data points shown in **(D)**.

**Figure 9:**
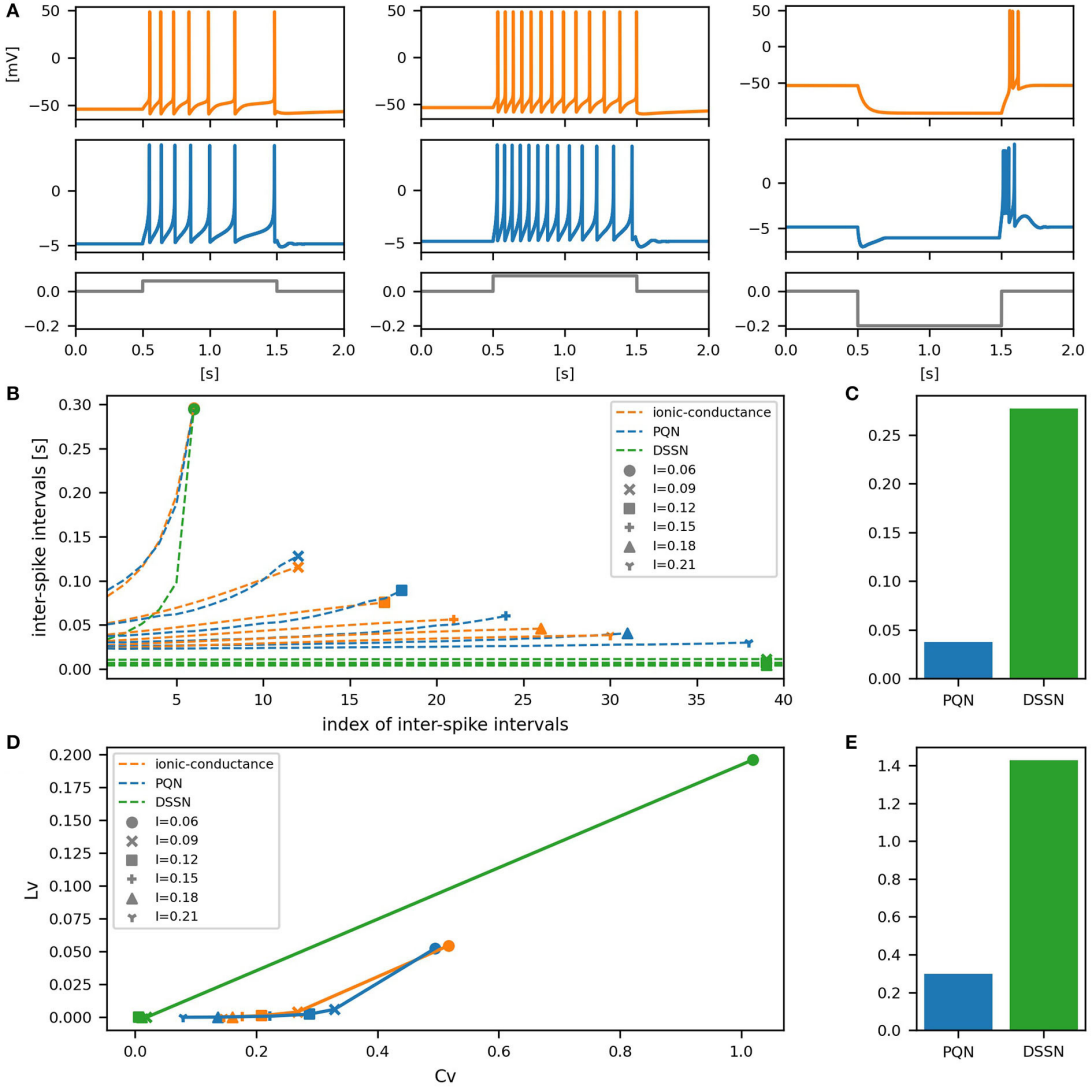
Waveforms and spiking properties in LTS mode. **(A)** Waveforms of the ionic-conductance model (orange) and the PQN model (blue). Gray lines represent stimulus inputs. **(B)** The transitions of ISIs are shown while increasing the magnitude of the stimulus input. **(C)** The mean square errors of the PQN model and the DSSN model with the ionic-conductance model over the data points shown in **(B)**. **(D)** Values of the *C*_*V*_ and *L*_*V*_ were calculated from the waveforms and plotted while varying the magnitude of the stimulus inputs. The waveforms used here are the same as that used in **(B)**. **(E)** The mean square errors of the PQN model and the DSSN model with the ionic-conductance model over the data points shown in **(D)**.

**Figure 10:**
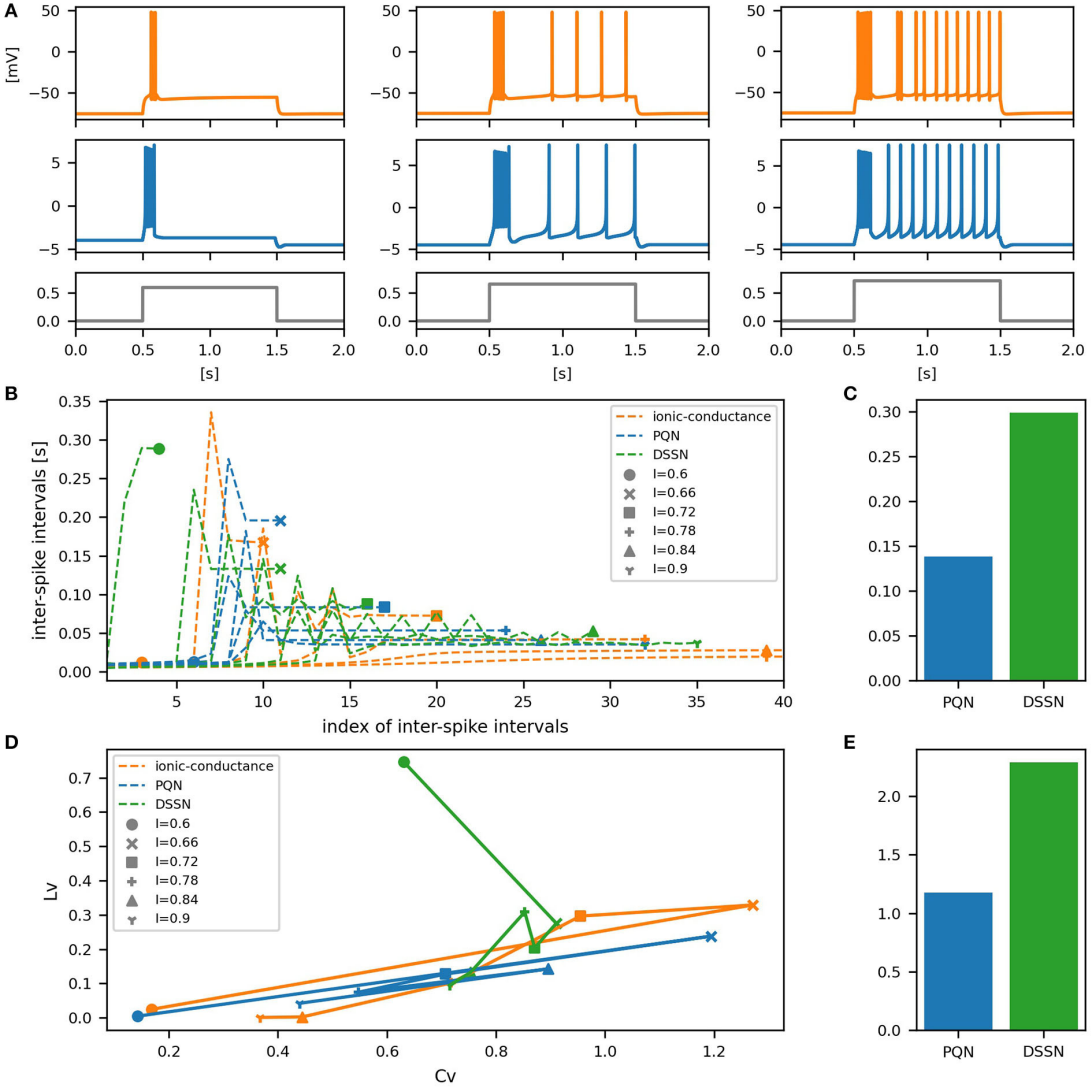
Waveforms and spiking properties in IB mode. **(A)** Waveforms of the ionic-conductance model (orange) and the PQN model (blue). Gray lines represent stimulus inputs. **(B)** The transitions of ISIs are shown while increasing the magnitude of the stimulus input. **(C)** The mean square errors of the PQN model and the DSSN model with the ionic-conductance model over the data points shown in **(B)**. **(D)** Values of the *C*_*V*_ and *L*_*V*_ were calculated from the waveforms and plotted while varying the magnitude of the stimulus inputs. The waveforms used here are the same as that used in **(B)**. **(E)** The mean square errors of the PQN model and the DSSN model with the ionic-conductance model over the data points shown in **(D)**.

**Figure 11:**
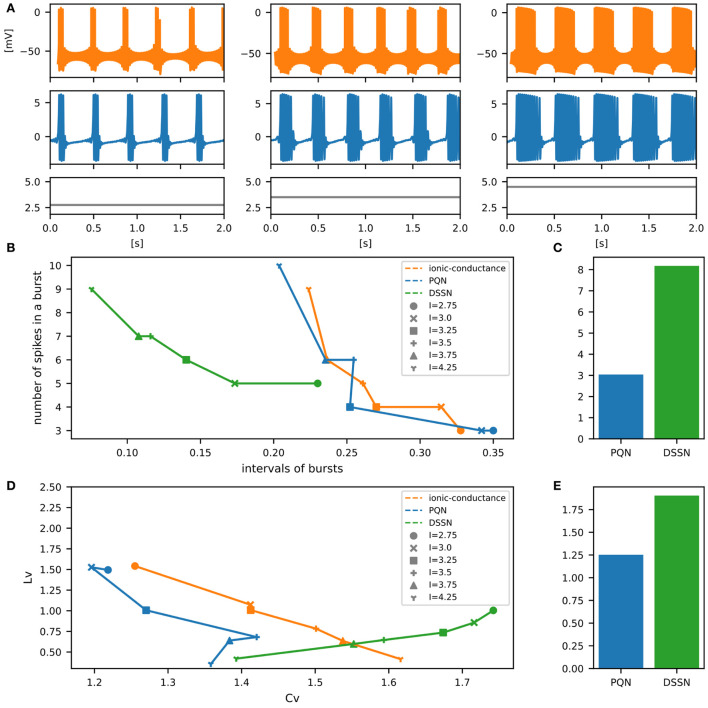
Waveforms and spiking properties in EB mode. **(A)** Waveforms of the ionic-conductance model (orange) and the PQN model (blue). Gray lines represent stimulus inputs. **(B)** Properties of bursting. The horizontal axis represents the interval between bursts, and the vertical axis represents the number of spikes in a burst. Stimulus inputs were increased by 0.25 from 2.75 to 4.25. **(C)** The mean square errors of the PQN model and the DSSN model with the ionic-conductance model over the data points shown in **(B)**. **(D)** Values of the *C*_*V*_ and *L*_*V*_ were calculated from the waveforms and plotted while varying the magnitude of the stimulus inputs. **(E)** The mean square errors of the PQN model and the DSSN model with the ionic-conductance model over the data points shown in **(D)**.

**Figure 12:**
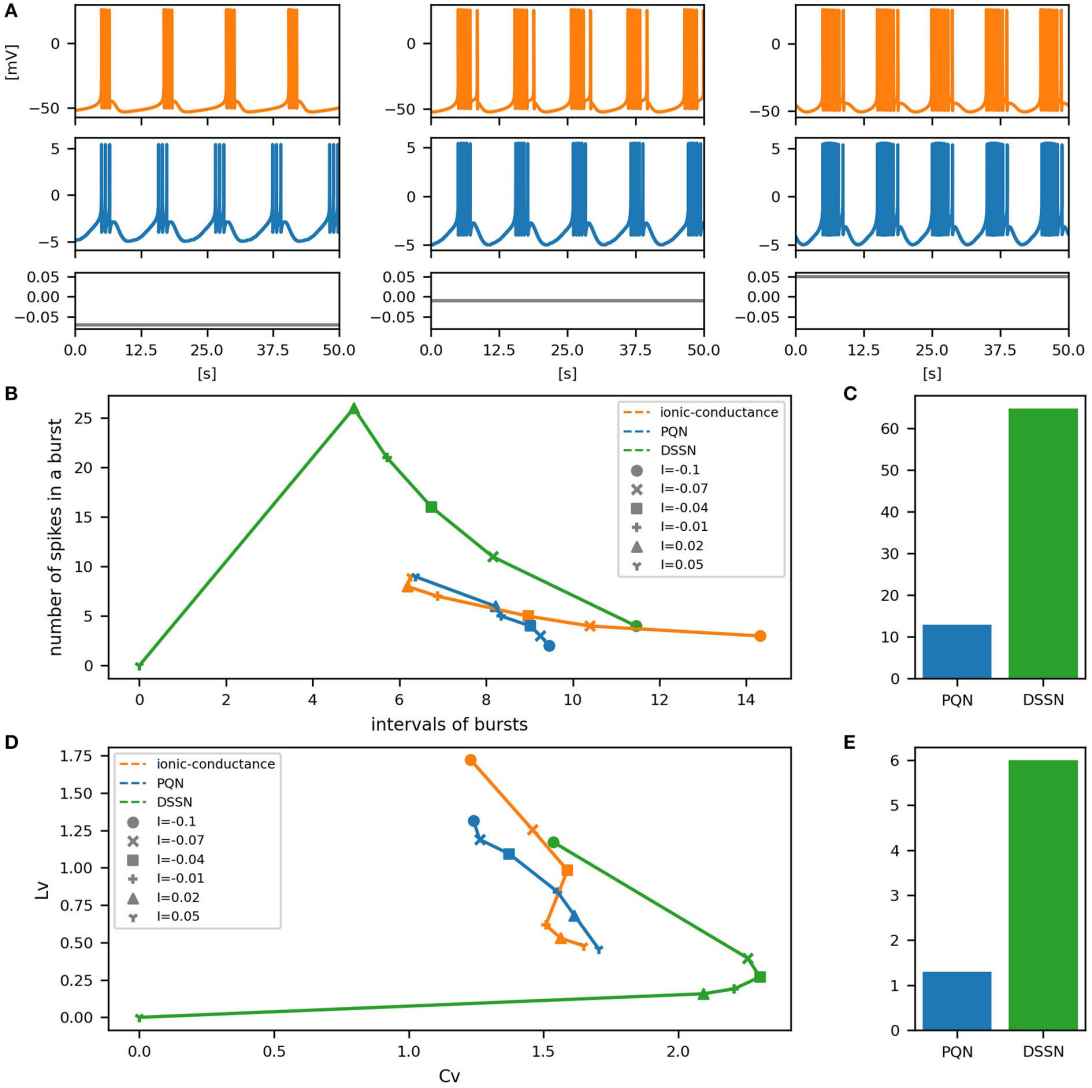
Waveforms and spiking properties in PB mode. **(A)** Waveforms of the ionic-conductance model (orange) and the PQN model (blue). Gray lines represent stimulus inputs. **(B)** Properties of bursting. The horizontal axis represents the interval between bursts, and the vertical axis represents the number of spikes in a burst. Stimulus inputs were increased by 0.25 from 2.75 to 4.25. **(C)** The mean square errors of the PQN model and the DSSN model with the ionic-conductance model over the data points shown in **(B)**. **(D)** Values of the *C*_*V*_ and *L*_*V*_ were calculated from the waveforms and plotted while varying the magnitude of the stimulus inputs. **(E)** The mean square errors of the PQN model and the DSSN model with the ionic-conductance model over the data points shown in **(D)**.

Figures 6–12A show the responses of the membrane potential in response to three different amplitudes of stimulus for excitatory RS, inhibitory RS, FS, LTS, IB, EB, and PB classes, respectively. The orange and blue plots represent the membrane potentials of the ionic-conductance and PQN models, respectively. The gray plots are the stimulus input currents, whose unit in the ionic-conductance model is pA. In the PQN model, they have no physical unit. In the excitatory RS mode, both models exhibit spike-frequency adaptation. Both models in the inhibitory RS mode show more intense spike-frequency adaptation than those in the excitatory RS mode. In the FS mode, weak spike frequency adaptation is only seen immediately after the step input is given. In the LTS mode, both models show a strong spike-frequency adaptation in response to positive step stimuli. Immediately after the long inhibitory input is removed, they show transient burst firing, which is called rebound bursting. Both models in the IB mode exhibit burst firing immediately after step stimuli are given. In response to more intense inputs, they show the periodic firings of the low frequency following the bursting. In the EB mode, both models repeat burst firings at constant intervals in response to constant stimulus inputs. As the stimuli become more intense, the number of spikes in the bursts becomes larger, and the intervals between bursts become shorter. Both models in the PB mode show repeated burst firings at constant intervals to the constant stimulus inputs. During the intervals between burst firings, the subthreshold oscillation is seen in the EB class but not in the PB class.

To quantitatively assess the differences in activities between the ionic-conductance model and the PQN and DSSN models, we employed several statistics. For the RS, FS, LTS, and IB classes we initially measured and plotted the transition of inter-spike intervals (ISIs) for each spike sequence (Figures 6–10B). The RS, FS, and LTS classes exhibit spike frequency adaptation, and the ISI slowly increases in response to constant input. We measured the transition of ISIs because it can capture the characteristics of the change in ISIs. In the IB class, the ISIs are small for the first few spikes due to the initial bursting, and then ISIs become larger. ISIs were also measured and plotted in the IB class to visualize these characteristics. The EB and PB classes repeat burst firing in response to a constant stimulus. The characteristics of repeated burst firing were quantified by the number of spikes in a single bursting and the intervals between bursting (Figures 11, 12B).

In addition, for all classes, we plotted the coefficient of variation (*C*_*V*_) and local variation (*L*_*V*_) (Shinomoto et al., 2003). The *C*_*V*_ and *L*_*V*_ are calculated as follows:


(42) $$\begin{array}{l} C_{V}=\sqrt{\frac{1}{n-1}\overset{n}{\underset{i=1}{\sum }}{\left(T_{i}-\bar{T}\right)}^{2}}/\bar{T}, \end{array}$$

(43) $$\begin{array}{l} L_{V}=\frac{1}{n-1}\overset{n-1}{\underset{i=1}{\sum }}\frac{3{\left(T_{i}-T_{i+1}\right)}^{2}}{{\left(T_{i}+T_{i+1}\right)}^{2}}, \end{array}$$

where *T*_*i*_ represents the *i*th ISI, and $\bar{T}$ is the average of *T*_*i*_. *n* corresponds to the number of spikes. The coefficient 3 in *L*_*V*_ is determined so that the expectation value of *L*_*V*_ in the Poisson spike sequence becomes one. The *C*_*V*_ represents the standard deviation divided by the mean ISI, and *L*_*V*_ measures a local fluctuation of the ISIs. Both *C*_*V*_ and *L*_*V*_ become zero for a spike sequence whose ISIs are constant. The *C*_*V*_ and *L*_*V*_ are widely used (Shinomoto et al., 2005a, 2009; Miura et al., 2006) to assess the firing properties of neurons in the nervous system.

In Figures 6–10B, the horizontal axes represent the index of the ISIs, and the vertical axes represent the length of the ISIs. Solid, dashed, and dotted curves represent the ionic-conductance model, PQN model, and DSSN model, respectively. Six different magnitudes of step inputs are given. Although the DSSN model reproduces the mathematical structure of each class, it is not intended to reproduce the transition of spiking activities in response to the change in stimulus magnitude. In these figures, the input to the DSSN model is linearly transformed so that the number of spikes in the DSSN model matches those in the ionic-conductance model when the weakest input is applied in each figure. The response of the DSSN model differs significantly from that of the ionic-conductance model, while the response of the PQN model is closer to the ionic-conductance model. Figures 6–10C show the mean square errors of the PQN model and the DSSN model with the ionic-conductance model over the data points shown in Figures 6–10B. The value of the mean square errors of the PQN model was about half of that of the DSSN model for the IB class and less than one-fifth for the other classes.

In Figures 11, 12B, the timing properties of bursting are plotted while increasing the magnitude of the stimulus input. The horizontal axes represent the intervals between the bursting phases, and the vertical axes represent the number of spikes in a burst. Six different magnitudes of constant inputs are given. The inputs to the DSSN model were linearly transformed so that the number of spikes in the DSSN model matches those in the ionic-conductance model when the weakest input shown in each figure is given. Figures 11, 12C show the mean square errors of the PQN model and the DSSN model with the ionic-conductance model over the data points shown in Figures 11, 12B. The value of the mean square errors of the PQN model was about a quarter of that of the DSSN model.

In Figures 6–12D, the characteristics of the three models are plotted on the *C*_*V*_-*L*_*V*_ plane. The response data used in Figures 6–12B were used here. Figures 6–12E show the mean square errors of the PQN model and the DSSN model with the ionic-conductance model over the data points shown in Figures 6–12D. The value of the mean square errors of the PQN model was about half of that of the DSSN model for the IB and EB classes and less than one-quarter for the other classes.

### 3.3. Resource utilization on the FPGA

Table 1 shows resource consumption of the PQN, DSSN, and ionic-conductance models in the FPGA implementation. Previous studies (Nanami et al., 2016, 2017, 2018) of the DSSN model showed only the resources of the circuit of the DSSN engine, which computes the values of the state variables in the next step from the current values of the state variables. Therefore, we show the resources of both the PQN engine and the PQN unit for comparison. Here, look-up tables (LUTs) are truth tables and digital signal processors (DSPs) are blocks for complex calculations. Flip-flops (FFs) are memory elements and are mainly used for the registers. In the PQN and DSSN models, LUTs are mainly used for addition, and the DSP is used to calculate square *v*. The consumption of LUTs in the PQN engines is larger than that of the DSSN engines, except for the PB mode. This is because we focused more on reproducing spiking properties than on reducing resources in parameter tuning. Both the PQN and DSSN models consume only one DSP. The PQN unit consists of the PQN engine, FIFO memories, and the controller part responsible for data transfer between the PQN engine and FIFO memories, requiring more LUTs and FFs than the PQN engine. The PQN unit operates at 100 MHz clock at maximum and is capable of simulating 9993 neurons in real-time. Since the time step of the PB mode is 1 ms, it can be estimated that a single PQN unit of the PB mode can compute 99,993 neurons, but due to the lack of capacity of the block RAM, only 16,384 neurons are realized here. The FPGA implementation (Khoyratee et al., 2019) of the ionic-conductance model is programmable and can cover RS, FS, LTS, and IB classes by varying parameters. However, it can only simulate 500 neurons and requires a large amount of DSPs, which can be a bottleneck in building large-scale networks.

**Table 1:** FPGA implementation results of the PQN, DSSN, and ionic-conductance models.

	**Mode**	**LUT**	**FF**	**DSP**		
PQN engine	Class *II*	526	485	4		
	RS exci	1,407	480	1		
	RS inhi	1,630	474	1		
	FS	1,500	463	1		
	LTS	2,060	590	1		
	IB	1,779	571	1		
	EB	1,843	486	1		
	PB	1,919	596	1		
DSSN engine (Nanami et al., 2016, 2017, 2018)	RS exci	697	109	1		
	RS inhi	690	109	1		
	FS	681	109	1		
	LTS	938	120	1		
	IB	1,265	136	1		
	EB	1,320	110	1		
	PB	3,318	228	1		
	**Mode**	**LUT**	**FF**	**DSP**	**N**	**F (MHz)**
PQN unit	Class *II*	783	819	4	9,993	100
	RS exci	1,771	963	1	9,993	100
	RS inhi	1,995	957	1	9,993	100
	FS	1,866	946	1	9,993	100
	LTS	2,458	1,111	1	9,993	100
	IB	2,177	1,092	1	9,993	100
	EB	2,210	969	1	9,993	100
	PB	2,089	1,094	1	16,384	100
ionic-conductance unit (Khoyratee et al., 2019)	RS, FS, LTS, and IB	2,360	5,551	28	500	100
Available (Xilinx Artix-7 XC7A35T)		20,800	41,600	90		

## 4. Conclusion

In this study, we proposed the PQN model, which is a refined version of the DSSN model. The PQN model has fewer parameters than the DSSN model, and its parameter sets for each class were renewed.

It was shown that the two-variable PQN model can properly reproduce the Class *II* in Hodgkin's classification. Both the graded response to pulse stimulus and the standard Type *II* PRC, which are typical in the Class *II* neurons, were confirmed. Note that these properties cannot be reproduced by the I&F-based models. For the other variants of the PQN model, parameters were fitted to reproduce the spiking activities in the ionic-conductance models of the RS, FS, LTS, IB, EB, and PB classes. With the RS, FS, LTS, and IB classes, we measured the transition of the ISIs of the spike sequences to evaluate characteristics of the spike-frequency adaptation and initial bursting with subsequent sparse firing. With the EB and PB classes, the bursting properties were evaluated by the interval between bursts and the number of spikes in a burst. In addition, statistical indicators for spike sequence classification in theoretical neuroscience (the *C*_*V*_ and *L*_*V*_) were evaluated for all classes. It is reported (Shinomoto et al., 2003, 2005b) that spike trains recorded from different areas of the cortex exhibit different values of *C*_*V*_ and *L*_*V*_. *C*_*V*_ focuses on variations in the length of ISIs across entire spike trains, while *L*_*V*_ captures the change in adjacent ISIs. For example, in the bursting neuron class including the EB and PB, *L*_*V*_ shows larger values than that in other classes because ISIs change extremely at the beginning and end of bursts. These statistics showed that the PQN model reproduces the activities of the ionic-conductance models with dramatically higher accuracy than the DSSN model.

In Table 1, the circuit resources required for updating the state variables for a single time step were compared between the PQN and DSSN models. Since a single DSP supports the multiplication of 25-bit by 18-bit, the Class *II* mode with 28-bit state variables requires four DSPs. The other modes require only one DSP as well as the DSSN model. The PQN model consumes a larger number of LUTs and FFs than the DSSN model. This is because the PQN model consumes more shifters and adders to accurately reproduce the activities of the ionic-conductance models. In addition, buffers in the pipeline process consume more FF in the PQN model. Please note that the implementation of the DSSN model was not pipelined in the previous studies (Nanami et al., 2017, 2018) because the main goal of these studies was to build parameter fitting methods of the model and the implementation was done only to verify that it worked correctly. Therefore, the maximum clock frequency and the number of neurons that can be run in the real-time simulation were not measured. The ionic-conductance unit consumes much more FFs than the PQN unit. Their implementation uses the coordinate rotation digital computer (CORDIC) algorithm to calculate hyperbolic functions, which is an iterative algorithm and requires long pipeline stages, thus consuming many FFs for buffers. In addition, the ionic-conductance model generally has more state variables, which also leads to an increase in FF consumption.

Table 2 shows resource utilization of the FPGA implementations of the IZH and AdEx models (Soleimani et al., 2012; Heidarpour et al., 2016; Heidarpur et al., 2019; Haghiri and Ahmadi, 2020). In Heidarpour et al. (2016) and Heidarpur et al. (2019), they used the coordinate rotational digital computer (CORDIC) algorithm to implement the quadratic and exponential terms. In Soleimani et al. (2012) and Haghiri and Ahmadi (2020), the differential equations are approximated by piecewise linear functions and power of 2-based functions to reduce circuit resources. All of these implementations are pipelined and can operate at clocks above 100 MHz. They do not require a DSP and consume much fewer LUTs than the PQN model. However, these IZH and AdEx models do not reproduce all the classes that the PQN models reproduced. They cover the RS, FS, and IB classes and also exhibit the rebound bursting seen in the LTS class. However, as far as we know, they do not cover the EB and PB classes. In addition, there is a significant gap between covering a certain class and faithfully reproducing the responses of the ionic-conductance model or an actual cell of that class.

**Table 2:** FPGA resource utilization of the IZH and AdEx models.

	**LUT**	**FF**	**DSP**	**F (MHz)**
IZH (Piecewise Linear-based) (Soleimani et al., 2012)	493	617	0	241.937
IZH (CORDIC-based) (Heidarpur et al., 2019)	229	410	0	183.4
AdEx (CORDIC-Based) (Heidarpour et al., 2016)	864	1,246	0	128.7
AdEx (Power-2 Based) (Haghiri and Ahmadi, 2020)	270	643	0	196

In order for the IZH and AdEx models to replicate the responses of the ionic-conductance models with inputs of various magnitudes, as the PQN model did here, extensive modification of parameters and linear or nonlinear transformations of the input stimuli are thought to be necessary. Furthermore, as reported in the previous study (Nanami and Kohno, 2016), the responses of the IB class of the IZH model differ significantly from those of the ionic-conductance model, whose dynamical structure of the fast subsystem varies depending on the value of the membrane potential. Therefore, in order to reproduce the IB class of the ionic-conductance model, the equation of the IZH model needs considerable modification. A previous study (Jolivet et al., 2004) demonstrated that an I&F-based model that has a quadratic term accurately reproduces the ionic-conductance model of the FS class. However, the FS is the class with a simplest dynamics. It is left unknown whether their approach can reproduce the activities of the other classes with more complex dynamical structures.

With the recent availability of a vast amount of anatomical and physiological data, data-driven SNN models (Markram et al., 2015; Bezaire et al., 2016; Ecker et al., 2020), which aim to create a complete copy of the mammalian brain, have been built. These data-driven SNN models consist of ionic-conductance models and are designed to faithfully reproduce target brain regions as precisely as possible. However, the enormous computational cost of the models precludes the casual use of the models and the construction of larger-scale networks. For example, the model (Bezaire et al., 2016) that reproduces a CA1 in a rat hippocampus runs on a supercomputer consisting of 3,000 processors and requires 1 h of execution time for a 1-s simulation. Since SiNNs using PQN models can dramatically reduce power consumption and downsize computational systems, without severely compromising the reproducibility of neuronal activities, the PQN model is expected to be a promising modeling tool for building data-driven SNNs, and it will also expand the applicability of data-driven SNNs in both scientific and engineering fields.

The ionic-conductance models of neurons in the Pre-Bötzinger complex has been studied (Butera et al., 1999a,b; Del Negro et al., 2001). These models exhibit activities of the square-wave bursting class. We will analyze the mathematical structure behind square-wave bursting and extend the PQN model to reproduce these models. In addition, we will examine efficient implementations of PQN models in the digital ASIC. The ASIC versions of PQN units can be expected to achieve higher power efficiency. Furthermore, we will study fully automatic parameter fitting methods applicable to arbitrary neuronal cells. In the PQN unit, all the parameters are configured as circuits of adders and shifters rather than data, thus a single PQN unit can simulate only a homogeneous population of neurons. We plan to modify the PQN unit to store a number of parameters as data so that it can simulate heterogeneous populations that belong to the same neuron class but have slightly different firing properties.

## Data availability statement

The original contributions presented in the study are included in the article/Supplementary material, further inquiries can be directed to the corresponding author.

## Author contributions

TN performed the study. TK supervised it. Both authors contributed to the article and approved the submitted version.

## Footnotes

^1^ https://github.com/tnanami/PQN_py

^2^ https://github.com/tnanami/PQN_vhdl
