# Supplementary material for: Piecewise quadratic neuron model: A tool for close-to-biology spiking neuronal network simulation on dedicated hardware
Source: Front Neurosci. 2023 Jan 9;16:1069133. doi: 10.3389/fnins.2022.1069133 (PMC9870328; doi:10.3389/fnins.2022.1069133)
Supplement: Supplementary file 1 [file Table_1.pdf]

frontiers

Supplementary Material, Piecewise Quadratic Neuron model : a tool for close-to-biology spiking neuronal network simulation on dedicated hardware

1 SUPPLEMENTARY NOTES

1.1 Detailed equations for digital circuit implementation

In digital arithmetic circuits, the equations are arranged for efficient implementation. For example, in the mode for the IB and LTS, the values of the next steps for each state variable are calculated as follows:

$$v_{\text{next}} = \begin{cases} v + v\_vv\_S + v\_v\_S + v\_n + v\_q + v\_I + v\_c & (v < 0) \\ v + v\_vv\_L + v\_v\_L + v\_n + v\_q + v\_I + v\_c & (v \geq 0), \end{cases} \tag{S1}$$

$$n_{\text{next}} = \begin{cases} n + n\_uS & (u < r_u) \\ n + n\_uL & (u \geq r_u), \end{cases} \tag{S2}$$

$$n_0 = \begin{cases} n\_vv\_S + n\_v\_S + n\_n + n\_c & (v < r_g) \\ n\_vv\_L + n\_v\_L + n\_n + n\_c & (v \geq r_g), \end{cases} \tag{S3}$$

$$q_{\text{next}} = \begin{cases} q + q\_vv\_S + q\_v\_S + q\_q + q\_c & (v < r_h) \\ q + q\_vv\_L + q\_v\_L + q\_q + q\_c & (v \geq r_h), \end{cases} \tag{S4}$$

$$u_{\text{next}} = u + u\_v + u\_u + u\_c, \tag{S5}$$

$$v\_vv\_S = Y_{v\_vv\_S}\; v^2, \tag{S6}$$

$$v\_vv\_L = Y_{v\_vv\_L}\; v^2, \tag{S7}$$

$$v\_v\_S = Y_{v\_v\_S}\; v, \tag{S8}$$

$$v\_v\_L = Y_{v\_v\_L}\; v, \tag{S9}$$

$$v\_n = Y_{v\_n}\; n, \tag{S10}$$

$$v\_q = Y_{v\_q}\; q, \tag{S11}$$

$$v\_I = Y_{v\_I}\; I_{\text{stim}}, \tag{S12}$$

$$v\_c = \begin{cases} \Delta t \frac{\phi}{\tau}(a_{fn} b_{fn}^2 + c_{fn} + I_0) & (v < 0) \\ \Delta t \frac{\phi}{\tau}(a_{fp} b_{fp}^2 + c_{fp} + I_0) & (v \geq 0), \end{cases} \tag{S13}$$

$$n\_uS = Y_{n\_uS}\; n_0, \tag{S14}$$

$$n\_uL = Y_{n\_uL}\; n_0, \tag{S15}$$

$$n\_vv\_S = Y_{n\_vv\_S}\; v^2, \tag{S16}$$

$$n\_vv\_L = Y_{n\_vv\_L}\; v^2, \tag{S17}$$

$$n\_v\_S = Y_{n\_v\_S}\; v, \tag{S18}$$

$$n\_v\_L = Y_{n\_v\_L}\ v, \tag{S19}$$

$$n\_n = Y_{n\_n}\ n, \tag{S20}$$

$$n\_c = \begin{cases} \Delta t\frac{\phi}{\tau}(a_{gn}b_{gn}^2 + c_{gn}) & (v < r_g) \\ \Delta t\frac{\phi}{\tau}(a_{gp}b_{gp}^2 + c_{gp}) & (v \geq r_g), \end{cases} \tag{S21}$$

$$q\_vv\_S = Y_{q\_vv\_S}\ v^2, \tag{S22}$$

$$q\_vv\_L = Y_{q\_vv\_L}\ v^2, \tag{S23}$$

$$q\_v\_S = Y_{q\_v\_S}\ v, \tag{S24}$$

$$q\_v\_L = Y_{q\_v\_L}\ v, \tag{S25}$$

$$q\_q = Y_{q\_q}\ q, \tag{S26}$$

$$q\_c = \begin{cases} \Delta t\frac{\phi}{\tau}(a_{hn}b_{hn}^2 + c_{hn}) & (v < r_h) \\ \Delta t\frac{\phi}{\tau}(a_{hp}b_{hp}^2 + c_{hp}) & (v \geq r_h), \end{cases} \tag{S27}$$

$$u\_v = Y_{u\_v}\ v, \tag{S28}$$

$$u\_u = Y_{u\_u}\ u, \tag{S29}$$

$$u\_c = -\Delta t\frac{\epsilon_u}{\tau}v_0, \tag{S30}$$

$$Y_{v\_vv\_S} = \Delta t\frac{\phi}{\tau}a_{fn}, \tag{S31}$$

$$Y_{v\_vv\_L} = \Delta t\frac{\phi}{\tau}a_{fp}, \tag{S32}$$

$$Y_{v\_v\_S} = -2\Delta t\frac{\phi}{\tau}a_{fn}b_{fn}, \tag{S33}$$

$$Y_{v\_v\_L} = -2\Delta t\frac{\phi}{\tau}a_{fp}b_{fp}, \tag{S34}$$

$$Y_{v\_n} = -\Delta t\frac{\phi}{\tau}, \tag{S35}$$

$$Y_{v\_q} = -\Delta t\frac{\phi}{\tau}, \tag{S36}$$

$$Y_{v\_I} = \Delta t\frac{\phi}{\tau}k, \tag{S37}$$

$$Y_{n\_uS} = \eta_0, \tag{S38}$$

$$Y_{n\_uL} = \eta_1, \tag{S39}$$

$$Y_{n\_vv\_S} = \Delta t\frac{\phi}{\tau}a_{gn}, \tag{S40}$$

$$Y_{n\_vv\_L} = \Delta t\frac{\phi}{\tau}a_{gp}, \tag{S41}$$

$$Y_{n\_v\_S} = -2\Delta t\frac{\phi}{\tau}a_{gn}b_{gn}, \tag{S42}$$

$$Y_{n\_v\_L} = -2\Delta t \frac{\phi}{\tau} a_{gp} b_{gp}, \tag{S43}$$

$$Y_{n\_n} = -\Delta t \frac{\phi}{\tau}, \tag{S44}$$

$$Y_{q\_vv\_S} = \Delta t \frac{\phi}{\tau} a_{hn}, \tag{S45}$$

$$Y_{q\_vv\_L} = \Delta t \frac{\phi}{\tau} a_{hp}, \tag{S46}$$

$$Y_{q\_v\_S} = -2\Delta t \frac{\phi}{\tau} a_{hn} b_{hn}, \tag{S47}$$

$$Y_{q\_v\_L} = -2\Delta t \frac{\phi}{\tau} a_{hp} b_{hp}, \tag{S48}$$

$$Y_{q\_q} = -\Delta t \frac{\phi}{\tau}, \tag{S49}$$

$$Y_{u\_v} = \Delta t \frac{\epsilon_u}{\tau}, \tag{S50}$$

$$Y_{u\_u} = -\Delta t \frac{\epsilon_u}{\tau} \alpha_u, \tag{S51}$$

where v_{next}, n_{next}, q_{next}, and u_{next}, are the value of the state variables in the next step. The Y_x, where x is v_vv_S, v_vv_L, v_v_S, v_v_L, v_n, v_q, v_I, n_uS, n_uL, n_vv_S, n_vv_L, n_v_S, n_v_L, n_n, q_vv_S, q_vv_L, q_v_S, q_v_L, q_q, u_v, or u_u, are the coefficients. They are calculated in advance and their multiplications with signals are implemented by shifters and adders. The signals v_vv_S, v_vv_L, v_v_S, v_v_L, v_n, v_q, v_I, n_uS, n_uL, n_vv_S, n_vv_L, n_v_S, n_v_L, n_n, q_vv_S, q_vv_L, q_v_S, q_v_L, q_q, u_v, u_u, and n_0 are the intermediate results of the calculation and are stored in registers for pipelined design. The v_c, n_c, q_c, and u_c, are constants, and they are calculated in advance and stored.

2 SUPPLEMENTARY TABLES

Table S1. Parameter set for the Class II mode.

Par.	Value	Par.	Value
Δ t	0.0001	τ	0.0064
a_{fn}	4	a_{fp}	-4
b_{fn}	-2	c_{fn}	0
a_{gn}	-3	a_{gp}	3
b_{gn}	-2	c_{gn}	-16
I_0	-16	k	8
ϕ	0.09375	r_g	-3

Table S2. Parameter set for the excitatory RS mode.

Par.	Value	Par.	Value
Δ t	0.0001	τ	0.0064
a_{fn}	1.5625	a_{fp}	-0.5625
b_{fn}	-1.125	c_{fn}	0
a_{gn}	1.0	a_{gp}	10.28125
b_{gn}	0.40625	c_{gn}	0
a_{hn}	0.28125	a_{hp}	9.125
b_{hn}	-7.1875	c_{hn}	-2.8125
I_0	2.375	k	36.4375
ϕ	4.75	ϵ_q	0.0693359375
r_g	0.0625	r_h	15.71875

Table S3. Parameter set for the inhibitory RS mode.

Par.	Value	Par.	Value
Δ t	0.0001	τ	0.0064
a_{fn}	1.4375	a_{fp}	-0.90625
b_{fn}	-1.0625	c_{fn}	0
a_{gn}	0.75	a_{gp}	14.1875
b_{gn}	0.625	c_{gn}	0
a_{hn}	0.0625	a_{hp}	13.0625
b_{hn}	-11.09375	c_{hn}	-1.65625
I_0	1.125	k	93.6875
ϕ	3.46875	ϵ_q	0.03515625
r_g	1.0625	r_h	0.40625

Table S4. Parameter set for the FS mode.

Par.	Value	Par.	Value
Δ t	0.0001	τ	0.0016
a_{fn}	1.9375	a_{fp}	-1
b_{fn}	-1.77734375	c_{fn}	0
a_{gn}	1.20703125	a_{gp}	15.76171875
b_{gn}	-0.9296875	c_{gn}	0
a_{hn}	0	a_{hp}	3.3046875
b_{hn}	-10.94921875	c_{hn}	0.74609375
I_0	-0.8984375	k	44.04296875
ϕ	0.3203125	ϵ_q	0.01611328125
r_g	-0.89453125	r_h	-3.6875

Table S5. Parameter set for the LTS mode.

Par.	Value	Par.	Value
Δ t	0.0001	τ	0.0016
a_{fn}	1.80859375	a_{fp}	-0.0048828125
b_{fn}	-1.1787109375	c_{fn}	0
a_{gn}	1.490234375	a_{gp}	7.4296875
b_{gn}	-0.5908203125	c_{gn}	0
a_{hn}	-0.103515625	a_{hp}	0.099609375
b_{hn}	2.361328125	c_{hn}	-0.048828125
I_0	-4.6455078125	k	16.076171875
ϕ	0.4794921875	ϵ_q	0.006591796875
r_g	0.7490234375	r_h	-0.619140625
ϵ_u	0.009765625	v_0	0
α_u	0.974609375	r_u	-6.5185546875
η_0	1.7509765625	η_1	1

Table S6. Parameter set for the IB mode.

Par.	Value	Par.	Value
Δ t	0.0001	τ	0.0008
a_{fn}	1.814453125	a_{fp}	-0.00390625
b_{fn}	-0.759765625	c_{fn}	0
a_{gn}	1.427734375	a_{gp}	1.18359375
b_{gn}	-0.046875	c_{gn}	0
a_{hn}	-0.208984375	a_{hp}	-0.4658203125
b_{hn}	1.8828125	c_{hn}	0.236328125
I_0	-6.1103515625	k	1.35546875
ϕ	0.4462890625	ϵ_q	0.00360107421875
r_g	-1.201171875	r_h	-0.6953125
ϵ_u	0.01654052734375	v_0	0
α_u	0.125	r_u	-31.6728515625
η_0	1.328125	η_1	1

Table S7. Parameter set for the EB mode.

Par.	Value	Par.	Value
Δ t	0.0001	τ	0.0064
a_{fn}	1.470703125	a_{fp}	-0.1181640625
b_{fn}	-1.0224609375	c_{fn}	0
a_{gn}	-1.92578125	a_{gp}	11.0087890625
b_{gn}	0.6708984375	c_{gn}	0
a_{hn}	6.5380859375	a_{hp}	-9.513671875
b_{hn}	-1.607421875	c_{hn}	12.787109375
I_0	-11.89453125	k	6.5234375
ϕ	0.7861328125	ϵ_q	0.0098876953125
r_g	2.71875	r_h	1.2421875

Table S8. Parameter set for the PB mode.

Par.	Value	Par.	Value
Δ t	0.001	τ	0.064
a_{fn}	1.9814453125	a_{fp}	-0.4521484375
b_{fn}	-0.9169921875	c_{fn}	0
a_{gn}	1.25	a_{gp}	16.0
b_{gn}	-0.2265625	c_{gn}	0
a_{hn}	-8.248046875	a_{hp}	14.658203125
b_{hn}	-2.259765625	c_{hn}	15.8720703125
I_0	-0.4609375	k	29.998046875
ϕ	3.12890625	ϵ_q	0.00494384765625
r_g	2.1953125	r_h	-0.943359375
ϵ_u	0.3043212890625	v_0	0.92578125
α_u	0.201171875		
